# The Effect of Selective Laser Trabeculoplasty on Intraocular Pressure in Patients with Dexamethasone Intravitreal Implant-Induced Elevated Intraocular Pressure

**DOI:** 10.1155/2020/3439182

**Published:** 2020-10-13

**Authors:** Amin Bennedjai, Vincent Theillac, Jad Akesbi, Raphaël Adam, Thibaut Rodallec, Chafik Keilani, Esther Blumen-Ohana, Antoine Labbé, Jean-Philippe Nordmann

**Affiliations:** ^1^Department of Ophthalmology 2, Quinze-Vingts National Ophthalmology Hospital, IHU FOReSIGHT, University Paris Descartes, Paris, France; ^2^Department of Ophthalmology 3, Quinze-Vingts National Ophthalmology Hospital, IHU FOReSIGHT, Paris Saclay University, Paris, France

## Abstract

**Introduction:**

To assess the safety and efficacy of selective laser trabeculoplasty (SLT) for ocular hypertension (OHT) induced by a dexamethasone (DEX) intravitreal implant.

**Materials and Methods:**

We performed a retrospective study of patients who underwent an SLT procedure for ocular hypertension induced by injection of a DEX intravitreal implant. Patients had, at least, one injection of the DEX-implant for symptomatic macular edema. SLT was delivered to 360° of the trabecular meshwork in two sessions. The primary outcome was a decrease in IOP, evaluated at one, three, and six months after the SLT procedure.

**Results:**

Twenty-six eyes of 22 patients were included. The mean intraocular pressure (IOP) measured after DEX-implant injection was 25.4 ± 5.4 mmHg, and the mean increase in IOP was 35.8 ± 14.6%. The mean follow-up after SLT was 18.3 ± 7.7 months. After SLT, the mean IOP dropped by 30.9% at one month (16.9 ± 4.5 mmHg, *p*=0.01), 33.6% at three months (16.0 ± 2.7 mmHg, *p* < 0.01), and 34.9% at six months (15.6 ± 2.1 mmHg, *p* < 0.01). Each patient had a minimum follow-up of 6 months after SLT. Eight eyes (31%) received a second DEX-implant injection after the SLT procedure without experiencing an increase in the IOP above 21 mmHg or >20%. No glaucoma surgery was required during the follow-up. The mean number of medications (1.65 ± 1.36) was significantly reduced at one (1.19 ± 1.20, *p*=0.04), three (0.96 ± 1.03, *p* < 0.01), and six months (0.77 ± 0.95, *p* < 0.01) after SLT.

**Conclusion:**

SLT is an effective and safe procedure to control OHT following DEX-implant intravitreal injection.

## 1. Introduction

Dexamethasone intravitreal implant 0.7 mg (Ozurdex®, Allergan, Dublin, Ireland) (DEX-implant) is a sustained-release corticosteroid device that is effective in the management of diabetic macular edema [1], age-related macular degeneration [2], retinal vein occlusions [3], noninfectious posterior uveitis [4], and resistant Irvine–Gass syndrome [5]. After DEX-implant injection, an increase in intraocular pressure (IOP) may be observed after 1.5 to 2.5 months [6] in 12.6% [7] to 36.0% [8, 9] of patients. Rajesh et al. reported that antiglaucoma medication or filtering surgery was needed for 91.8% and 3.1% of patients exhibiting a postinjection increase in intraocular pressure (IOP), respectively [10]. Similarly, 3% of patients treated with a DEX-implant for Irvine–Gass syndrome were eventually injected with anti-VEGF because of ocular hypertension (OHT), despite maximal medical antiglaucoma treatment [5]. After three or more injections, the frequency of mild OHT increased to 53% versus 31% after the first injection [11]. Moreover, the risk of a large increase in IOP is greater for patients with primary open-angle glaucoma (POAG) or suspected glaucoma at the time of DEX implantation [12].

The physiopathology of glucocorticoid-induced OHT and glaucoma may be related to the impairment of outflow through the trabecular meshwork (TM) via an enzymatic pathway responsible for mucopolysaccharide accumulation in the iridocorneal angle [13]. A mouse model has suggested a role for glucocorticoid receptor transactivation in regulating glucocorticoid-mediated gene expression in the TM [14]. Steroids may also have an effect on the TM extracellular matrix due to altered rates of protein synthesis or degradation or a combination of the two [12].

Selective laser trabeculoplasty (SLT) uses a 532 nm Q-switched, frequency-doubled Nd:YAG laser that delivers a short pulse (3 nanoseconds) [15]. It prevents heat dissipation outside of pigmented TM cells and does not cause collateral damage [15]. Selective laser trabeculoplasty is effective in lowering IOP in open-angle glaucoma [16–18] and can be offered as a first-line treatment for POAG or OHT [19]. SLT induces biological changes that modulate increased aqueous outflow through the TM, including changes in cytokine and interleukin-8 (IL-8) secretion and TM remodeling, which are affected by glucocorticoids [20]. Selective laser trabeculoplasty is also effective in treating OHT after intravitreal triamcinolone acetonide injection [21], as well as in preventing an increase in IOP when performed before injection [22].

Various guidelines establishing the management of OHT after intravitreal injection have been published [23]. Topical treatment is the first approach proposed, followed by surgical treatment in case of refractory OHT. The exact role of SLT in the algorithm of treatment of OHT after DEX-implant injection has not been yet determined. Moreover, no studies have evaluated the effect of SLT in patients with DEX-implant-induced OHT. Thus, the main objective of the present study was to evaluate the effectiveness and safety of the SLT procedure on increased IOP after DEX-implant injection.

## 2. Patients and Methods

This study was conducted at the Quinze-Vingts National Ophthalmology Hospital, Paris, France, in accordance with the tenets of the Declaration of Helsinki. We retrospectively reviewed the medical records of all patients who underwent an SLT procedure for uncontrolled OHT induced by DEX-implant injection between November 2017 and October 2018.

The study included patients who had, at least, one injection of the DEX-implant for symptomatic macular edema. All patients were symptomatic, and diagnosis of macular edema was established based on examination of the fundus and macular optical coherence tomography (OCT) showing a central macular thickness (CMT) of >300 *μ*m (Spectralis HRA + OCT, Heidelberg engineering Inc, Heidelberg®, Germany). Patients with uncontrolled IOP (according to their target IOP) despite maximal tolerated topical treatment three months after DEX-implant injection underwent the SLT procedure.

### 2.1. Injection Technique

An implant of 0.7 mg dexamethasone (Ozurdex®, Allergan, Irvine, CA) was inserted into the vitreous cavity through the pars plana at a distance of 3.5 mm from the limbus in pseudophakic eyes and 4 mm in phakic eyes after topical anesthesia (oxybuprocaine 1.6 mg/0.4 ml, Thea®, Clermont-Ferrand, France) and sterilization of the ocular and periocular surface with 5% povidone iodine. Patients were seen, at least, one, three, and six months after the injections, allowing measurement of the best corrected visual acuity (BCVA), IOP, CMT, and examination of the fundus.

### 2.2. SLT Procedure

All SLT procedures were performed, after topical anesthesia (oxybuprocaine), by trained physicians of our group with the same machine (TangoTM, EllexInc, Adelaide, Australia) using a Latina SLT lens (Ocular Instruments, Bellevue, Washington, USA) to visualize the TM. Selective laser trabeculoplasty was delivered to 360° of the trabecular meshwork in two sessions, one week apart, with the total number of nonoverlapping impacts varying from 80 to 110. The energy setting was from 0.8 to 1.2 mJ. One drop of 0.1% Indocollyre (Indometacine, Bausch & Lomb, Berlin, Germany) and 0.5% Iopidine (Alcon Laboratories, Inc., Ft. Worth, TX) were immediately administered after the procedure and for five days, three times a day, for Indocollyre.

### 2.3. Study Population

Each patient underwent a standardized examination, performed in our retinal disease department, with measurement of the BCVA in Early Treatment Diabetic Retinopathy Study (ETDRS) letters converted to LogMAR (Minimum Angle of Resolution) [24], IOP measurement using a Goldman applanation tonometer, examination of the fundus, and macular OCT. Every patient had an open angle on the Schaffer scale at gonioscopy examination. Open-angle glaucoma risk factors and the date and number of DEX-implant injections, the date, number, and characteristics of the SLT procedures, number and type of antiglaucoma medications, and surgical history were recorded.

### 2.4. Statistical Assessment

All statistical analyses were performed using GraphPad Prism 7® for Windows® (GraphPad Software, La Jolla, CA, USA). The primary outcome was a decrease in IOP evaluated at one, three, and six months after the SLT procedure. Quantitative variables were compared using the Student *t*-test. Survival curves were generated using the Kaplan–Meier method. Double-sided *p* values <0.05 were considered statistically significant.

## 3. Results

The study included 26 eyes of 22 patients. Baseline demographics and ocular parameters are presented in Table 1.

**Figure 1:**
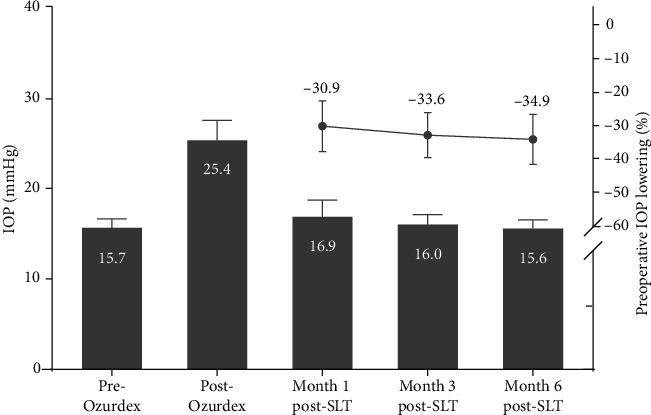
Intraocular pressure lowering after the SLT procedure with a follow-up of 6 months.

Best corrected visual acuity significantly improved following DEX-implant injection from 0.58 ± 0.42 to 0.32 ± 0.39 at six months after injection (*p*=0.01). The mean time between DEX-implant injection and the diagnosis of OHT was 55.8 ± 27.9 days. The SLT procedure was performed at a mean of 96.71 ± 14.73 days after DEX-implant injection. The mean duration of follow-up after SLT was 18.3 ± 7.7 months. The maximal IOP measured after DEX-implant injection was 25.4 ± 5.4 mmHg, with an increase in IOP of 35.8 ± 14.6%. After SLT, the mean IOP dropped by 30.9% at one month (16.9 ± 4.5 mmHg), 33.6% at three months (16.0 ± 2.7 mmHg), and 34.9% at six months (15.6 ± 2.1 mmHg) after SLT, showing a persistent and significant decrease in IOP (*p* < 0.01 compared to the pre-SLT IOP at each visit) (Figure 1). The SLT procedures are described in Table 2.

**Figure 2:**
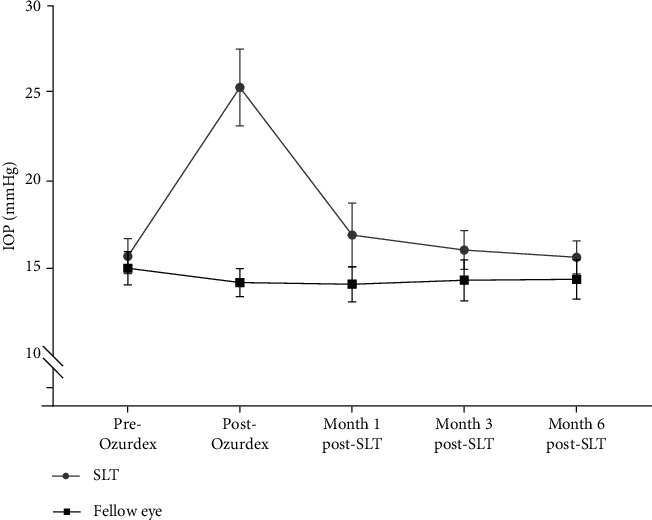
Evolution of mean intraocular pressure (IOP) before and after a DEX-implant injection in eyes that underwent selective laser trabeculoplasty versus contralateral eyes at one, three, and six months after treatment.

**Figure 3:**
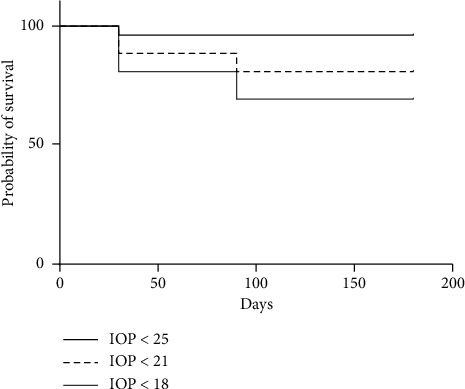
Kaplan–Meier survival curves plotting the cumulative probabilities that the IOP remains below 25 mmHg, 21 mmHg, and 18 mmHg, respectively, following the SLT procedure.

Eight eyes (31%) underwent a second DEX-implant injection after the SLT procedure without experiencing an increase in IOP (>20%) after one week or one, three, or six months of follow-up. Seven eyes did not require additional antiglaucoma drugs. The mean peak IOP after reinjection for patients who had SLT was 18.8 ± 1.5 mmHg, lower than the peak IOP after the first injection for patients requiring reinjection (27.3 ± 1.8 mmHg, *p*=0.01). The mean IOP values for the eyes that underwent SLT versus those of the contralateral eyes during the follow-up period are shown in Figure 2. During follow-up, 96.2%, 80.8%, and 69.2% of the treated eyes showed an IOP below 25, 21, and 18 mmHg, respectively (Figure 3).

The mean number of medications (1.65 ± 1.36) was significantly lower at one (1.19 ± 1.20, *p*=0.04), three (0.96 ± 1.03, *p* < 0.01), and six months (0.77 ± 0.95, *p* < 0.01) after trabeculoplasty. No oral treatment or surgery was required during the follow-up. Six of the 26 patients (23%) required antiglaucoma eye drops after six months, with a mean number of 0.5 ± 0.88 topical treatments, without oral medication or surgery.

Adverse effects after SLT included a moderate transient anterior chamber reaction for one patient (4%) and traumatic keratitis for another (4%), which healed after one-week of topical treatment (Tobradex® (0.3% tobramycin, 0.1% dexamethasone; Alcon Laboratories Inc., Fort Worth, Texas) three times a day and vitamin A ointment associated with artificial tears, respectively).

## 4. Discussion

The SLT procedure appears to be a valuable and safe tool to manage increased IOP after DEX-implant injection. The exact mechanism by which SLT decreases IOP has not been yet fully elucidated. It may involve macrophage activation, resulting from increased chemokine production, allowing TM clearing. The SLT procedure may also allow the TM to release factors that regulate the permeability of Schlemm canal endothelial cells [25]. This effect mirrors the physiopathology of steroid-induced OHT, which involves increased responsiveness of the membranes of goniocytes to steroids, leading to increased production of fibroblasts in the TM and resulting in aqueous retention [12]. The TM extracellular matrix may be remodeled by the expression of stromelysin-1 (MMP-3), resulting in an increase in aqueous outflow [26]. Selective laser trabeculoplasty appears to have some clinical efficacy in secondary glaucoma patients, especially when dysfunction of the TM is involved, such as in DEX-implant-induced OHT. Here, we report a larger decrease in IOP after SLT than previously reported after SLT performed for pigmentary glaucoma (14.5%) or pseudoexfoliation glaucoma (16.6%) [27].

This study highlights the potential interest of SLT after steroid-induced OHT. This procedure may be an alternative to the usual treatment, which involves topical ocular antiglaucoma medications. Two studies have already reported efficacy of the SLT procedure in steroid-induced OHT. Rubin et al. reported the efficacy of SLT, with a significative decrease of IOP (*p* < 0.007) in five of seven patients [21]. Bozkurt et al., showed that the increase in IOP after intravitreal triamcinolone acetonide injection may be prevented by performing SLT if the baseline IOP is >21 mmHg [22]. The SAFODEX study demonstrated that OHT can be observed for 28.5% of DEX-implant injected eyes [28]. A patient who experiences OHT after the first injection has a significant risk of experiencing an increase in IOP after reinjection. The frequency of OHT (>23 mmHg) following one, two, or three reinjections is 31%, 26%, and 53%, respectively [11]. In the present study, none of the eight eyes that underwent DEX-implant reinjection after the SLT procedure experienced a major peak of IOP after SLT. These results suggest that the SLT procedure may also be useful in preventing new episodes of OHT after reinjection of the DEX-implant in patients who already experienced steroid-induced OHT.

Filtering surgery is more often needed to control corticosteroid-induced OHT in patients with branch or central retinal vein occlusion (RVO). Ocular hypertension in these patients may be multifactorial and the associated retinal ischemia may be responsible for persistent OHT in RVO [6]. Of note, in our study, the SLT procedure was also effective in controlling corticosteroid-induced OHT for patients with RVO, as the IOP of treated eyes tended to decrease to the same level as that of the adelphic eyes after three to six months.

Our study had several limitations, notably its being a retrospective study with a limited number of patients. Nonetheless, SLT can be considered to lower DEX-implant-induced increases in IOP. Selective laser trabeculoplasty may be a safe and effective alternative to antiglaucoma eye drops as a first-line treatment and probably as a prophylactic procedure to avoid peak IOP in patients presenting corticosteroid-induced OHT.

## Figures and Tables

**Table 1:** Baseline demographic characteristics of the 22 patients who underwent SLT after DEX-implant injection.

Patient characteristics (*n* = 22)	
Male	12 (55)
Female	10 (45)
Caucasian	18 (82)
African	4 (18)
Age, mean ± SD	69.6 ± 15.4
Family history of glaucoma	2 (9)

Eyes characteristics (*n* = 26)	
Retinal disease	
Diabetic macular edema	15 (57)
Irvine–Gass syndrome	8 (31)
Branch retinal vein occlusion	3 (12)
Cup-to-disc ratio ± SD	0.38 ± 0.19
Pseudophakic	19 (73)
PPV	5 (19)
Retinal laser	8 (31)
PPRP	7 (27)
Focal laser	1 (4)

The results are presented as *n* (%) for categorical variables. SD, standard deviation; PPV, pars plana vitrectomy; PPRP, peripherical pan-retical photocoagulation.

**Table 2:** Characteristics of the SLT procedures.

Parameters of the SLT procedures	
Total number of SLT procedures	26
Right eyes	10
Left eyes	16
Mean number of spots ± SD	91.6 ± 20.2
Total energy (mJ) ± SD	75.9 ± 24.4
New DEX-implant after SLT	8

## Data Availability

The paper data used to support the findings of this study are available from the corresponding author upon request.
